# Initial Feasibility of a Woman-Focused Intervention for Pregnant African-American Women

**DOI:** 10.1155/2011/389285

**Published:** 2011-03-23

**Authors:** Hendrée E. Jones, Nancy D. Berkman, Tracy L. Kline, Rachel Middlesteadt Ellerson, Felicia A. Browne, Winona Poulton, Wendee M. Wechsberg

**Affiliations:** Substance Abuse Treatment Evaluation and Intervention, RTI International, 3040 Cornwallis Road, Research Triangle Park, NC 27709, USA

## Abstract

African-American women who use crack are vulnerable to HIV because of the complex social circumstances in which they live. Drug-abuse treatment for these women during pregnancy may provide time for changing risk behaviors. This paper examines the initial 6-month feasibility of a women-focused HIV intervention, the Women's CoOp, adapted for pregnant women, relative to treatment-as-usual among 59 pregnant African-American women enrolled in drug-abuse treatment. At treatment entry, the women were largely homeless, unemployed, practicing unsafe sex, and involved in violence. Results indicated marked reductions in homelessness, use of cocaine and illegal drugs, involvement in physical violence, and an increase in knowledge of HIV from baseline to 6-month followup for both conditions. Findings suggest that the Women's CoOp intervention could be successfully adapted to treat this hard-to-reach population. Future studies should examine the efficacy of the pregnancy-adapted Women's CoOp for women not enrolled in drug-abuse treatment.

## 1. Introduction

The risk of contracting HIV is one of the most devastating health threats African-American women who use crack cocaine face. HIV prevalence rates among African-American women range from a low rate of 1.7% among noninjecting drug users who do not trade sex to a high rate of 54% among homeless African-American women, many of whom trade sex for drugs and survival items [1–5]. Crack cocaine use also has been repeatedly associated with increased sexual activity; if the sex is unprotected, unplanned pregnancies and HIV can result [6, 7]. 

African-American women who use crack are vulnerable to HIV because of the complex social circumstances in which they live. These social circumstances may produce situations where these women engage in multiple high-risk HIV behaviors [8]. Furthermore, the combination of crack, alcohol use, and sexual-risk behaviors (e.g., trading sex for drugs or survival items, inconsistent condom use, multiple partners) place African-American women at greater risk for HIV infection than other drug-using groups [9]. 

Many of these crack-using African-American women lack self-sufficiency, rely on public assistance for long durations, are often unstably housed, experience repeated episodes of homelessness, have no or scanty employment records, lack education and job skills, and live in poverty [9–15]. The lives of substance-using African-American women also are often characterized by inflicting or being a victim of violence, crime, childhood and current sexual, physical, and emotional victimization, as well as symptoms of anxiety, depression, and posttraumatic stress disorder [9, 16–18].

Many of these African-American women's struggles against social, cultural, and economic subordination include survival strategies that have the burden of drug use, sexual risk, HIV risk, homelessness, and violence [19]. In the context of this inequality, many impoverished African-American women also face unplanned pregnancies [20] and poor pregnancy outcomes [21]. In all but the rarest cases, licit and illicit substance-use starts well before pregnancy [22]. Drug use during pregnancy is associated with a multitude of adverse maternal, fetal, and neonatal outcomes, which occur in the context of poverty and psychosocial and environmental concerns. Substance-using pregnant African-American women tend to have completed approximately 11 years of education, be unmarried, have a history of substance-use in their families, have a history of physical and sexual victimization, have significant health and/or mental health problems (e.g., depression and/or anxiety), have current problems with criminal justice, have current and/or past involvement with Child Protective Services, lack stable housing, and be unemployed with poor vocational training [23–26]. As such, pregnant African-American women with substance use disorders are a very vulnerable population with meager support networks and few, if any, resources [27]. Yet they are often the targets of prejudicial and judgmental treatment by health care providers and coercive policies implemented by child welfare systems, juvenile/family courts, and the criminal justice system [28, 29]. 

 These barriers frequently deter African-American women with substance-use disorders from seeking concurrent prenatal care and substance use disorder treatment. The social, cultural, and economic subordination that African-American women face set the stage for a lack of prenatal care and an array of medical and obstetrical complications, reducing the chances of a healthy pregnancy outcome regardless of the effects of the drug(s) they are abusing. These adverse effects can be even further compounded if the pregnant woman has HIV. Thus, African-American women who are using crack cocaine are in critical need of an effective HIV risk-reduction intervention that acknowledges the multiple adverse influences in their lives, addresses their specific barriers to success, and empowers them to make positive choices in their lives for themselves and their children. 

The most effective HIV risk-reduction interventions for women are based on social psychological theory and are women-focused, culturally sensitive, and incorporate women-only sessions led by peers [30]. The Women's CoOp is an intervention that has resulted in HIV-risk behavior reduction in African-American women, and it has shown consistent reductions in homelessness and increases in employment as well as in condom use [30]. Given these improvements in risk behavior among nonpregnant African-American women, it was important to see if this intervention could be adapted for pregnant African-American women who, once pregnant, are at increased vulnerability for the consequences of HIV risk behavior, violence, homelessness, and unemployment [31]. 

### 1.1. Purpose of the Present Study

The purpose of the study is twofold. First, we will describe the multifaceted HIV risk behaviors and the psychosocial service needs of a sample of African-American pregnant women enrolled in drug-abuse treatment. Second, we will examine the initial feasibility of an adapted women-focused HIV intervention, Women's CoOp for Pregnant African-American Women, relative to treatment-as-usual for drug-abusing, pregnant African-American women in treatment. The intervention and the process to adapt it are described elsewhere [31]. This paper summarizes the results of the 6-month outcomes, the final time point of evaluation.

## 2. Methods

### 2.1. Study Design

A small-scale randomized clinical trial with pregnant African-American women in drug-abuse treatment was begun in May 2007 to measure the feasibility of the Women's CoOp for Pregnant African-American Women intervention relative to treatment-as-usual in multiple areas (e.g., substance abuse, HIV risk, homelessness, employment, intimate partner violence, and birth outcomes. This study was approved by the IRB of RTI International. Because the procedures of this study have been reported elsewhere [31]; a full summary of procedures is not provided here.

### 2.2. Outreach

Participants were recruited through targeted outreach, regional drug treatment centers, radio advertisements, and referrals from health department officials in North Carolina. Any out-of-treatment women were first referred to drug-abuse treatment. After drug-abuse treatment entry, these women could be enrolled in the study.

### 2.3. Eligibility Criteria

Eligibility criteria included being female, at least 18 years of age, self identified as Black/African-American, at least 14 weeks and not more than 32 weeks pregnant (pregnancy determined by urine testing and self reported gestational age based upon last menstrual period), and currently enrolled in a drug-abuse treatment program. Participants also needed to self-report use of an illicit drug within the past year. Additional eligibility criteria included being willing and with the cognitive capacity to provide written informed consent and being willing to provide verifiable locator information for followup assessments [31].

### 2.4. Recruitment

Women were recruited between May 2007 and February 2009. In total, 96 women were screened for the study, of whom 37 were found to be ineligible, including treatment refusals (*n* = 23), lack of self-reported use of any illicit drug in the past year (*n* = 21), outside the gestation window (*n* = 12), and not being pregnant (*n* = 6). Thus, 59 women from 11 different drug-abuse treatment programs were randomized into one of two study conditions, either the Women's CoOp for Pregnant African-American Women (*n* = 30) or treatment-as-usual (*n* = 29) after their baseline assessment [31]. 

### 2.5. Data Collection

At both baseline and followup assessments, participants were asked to complete a urine drug screen for methamphetamine, amphetamine, cocaine, heroin, marijuana, and ecstasy and an audio computer-assisted self-interview (ACASI) that included demographic (baseline only) and health-related questions regarding substance use, sexual-risk behaviors, experiences with violence, and prenatal care [31]. Followup interviews were then collected at 3 months (data not analyzed or shown due to amount of missing data) and 6 months following baseline assessment, at which time 85% of the sample was assessed.

### 2.6. Intervention Conditions

#### 2.6.1. The Women's CoOp for Pregnant African-American Women

The Women's CoOp for Pregnant African-American Women in drug-abuse treatment was developed from the original Women's CoOp that focuses on preventing HIV through drug use and sexual-risk reduction, improving relationships with men and social support, and receiving HIV testing [30]. Behavioral skills training (e.g., male and female condom demonstration and practice), roleplay to improve negotiation and communication skills, and personalized risk-reduction plans are key elements of the intervention [30]. Women's CoOp for Pregnant African-American Women retained the core elements of the Women's CoOp, and also included material on the risks of using drugs while pregnant, parenting, violence and victimization, and use of antiretroviral therapy (ART). The program also used short video vignettes of African-American women to emphasize key points [31]. 

#### 2.6.2. Treatment-As-Usual Control Group

The control group received no intervention beyond what they received as a part of the treatment program in which they were enrolled. 

### 2.7. Measures

#### 2.7.1. The Revised Risk Behavior Assessment (RRBA)

The RRBA has 10 sections, which contain questions about demographics and social characteristics; health knowledge; alcohol use; drug use; drug injecting; sexual practices; power and empowerment; conflict and victimization (stigma and vulnerability); physical and mental health and HIV. 

Although the RRBA has excellent psychometric properties for African-American women [9], an adaptation was necessary to measure particular concerns related to pregnant women. Based on a review of the literature and consultation with experts, several new questions were added. Specifically, experiences with personal violence were assessed by asking the participant how many times in the past 90 days she behaved in a violent manner toward others, including insulting or swearing at someone, beating up someone, and using a knife or gun on someone (Cronbach's Alpha = 0.91). These items were condensed into an indication of violent behavior in the past 90 days. Moreover, a 22-item scale adapted from the RRBA assessed participants' knowledge about HIV and STD transmission, drug and alcohol use, and substance-use influence on fetal development (Cronbach's Alpha = 0.71). These items were combined into a total HIV and drug risk knowledge score with a range of scores from 0 to 22.

An ACASI version was administered to insure privacy for respondents, given the inclusion of more intimate violence measures. 

#### 2.7.2. Urine Drug Testing

Urine drug testing for methamphetamine, amphetamine, cocaine, heroin, marijuana, and ecstasy was conducted at baseline and followup using a rapid 6-panel urine dip test.

## 3. Analysis

Analytic methods began with descriptive statistics on demographic variables of interest (e.g., age, employment status, marital status, homeless status), followed by inferential analyses on the outcome variables of interest using *t*-tests on continuous data to compare the Women's CoOp for Pregnant African-American Women and treatment-as-usual conditions within time and chi-squared analyses for dichotomous and ordinal data. Subscale items combined into an additive or averaged scale score were assessed through Cronbach's Alpha reliability to check internal consistency. Only those scales with an acceptable statistic (*α* = 0.70 or greater) were included in the analyses. In addition to the descriptive and reliability analyses, change-over-time assessments were conducted for the substance-use, sexual-risk, and violence outcomes of interest using the paired *t-*test for continuous data, the McNemar test for dichotomous data, and the Wilcoxon signed-ranks test for ordinal data. All analyses were conducted using the SAS 9.2 statistical software. Because both the Women's CoOp for Pregnant African-American Women and treatment-as-usual addressed the importance of substance-use reduction, an overall reduction in drug and alcohol use over time in both intervention conditions was expected.

## 4. Results

### 4.1. Participant Demographics and Characteristics

Table 1 shows that on average, randomized participants were in their late twenties, and a majority had less than a high school education, had never married, and reported that this pregnancy was unplanned. The average gestational age at study entry was 24 weeks (SD = 7.0). Women reported being an average of 10 weeks pregnant (SD = 7.0) at the time of pregnancy awareness, 88% reported having received at least some prenatal care, and 97% reported planning to keep the baby after delivery (some data are not shown in Table 1). Of note, the Women's CoOp for Pregnant African-American Women and treatment-as-usual conditions did not significantly differ at baseline on any of the measures found in Table 1 or reported in the text.

An examination of baseline characteristics of women who completed 6-month followup surveys indicates the severe social circumstances that the sample experienced when they entered this study (Table 2). More than 40% were homeless, and more than 75% were unemployed; 18% reported that they had an alcohol problem, and 58% reported that they had a drug problem. On a positive note, the participants had good knowledge of the risks of HIV and the effects of prenatal exposure to drugs of abuse, correctly answering, on average, 18 of the 22 questions. However, in the past 90 days, 33% of women who had sex indicated that they had never used a condom, and 62% indicated that they had been in violent situations in which they were violent toward someone else. Nonetheless, in both cases, more than 75% of the sample reported that they want to stop/reduce using drugs for themselves, and 80% wanted to stop/reduce drug use to have a healthy baby, indicating substantial motivation on the part of the participants as they participated in drug-abuse treatment. Motivation for reducing or stopping drug use also was found in over half of the women in the sample, who were fearful that Child Protective Services would take their child away if they did not change their drug use behavior.

Finally, it should be noted that the Women's CoOp for Pregnant African-American Women and treatment-as-usual conditions did not significantly differ at baseline on any of the outcome measures found in Table 2 (all *P*s > .17).

### 4.2. Intervention Evaluation

There were marked reductions in homelessness in both groups at 6-month followup (see Table 2), suggesting that the women were finding stable housing. Use of cocaine and illegal drugs dropped markedly, as did threats of physical violence toward others. Further, on average, the participant's knowledge about HIV and the risks of prenatal drug exposure increased, showing improvement from baseline assessment to 6-month followup. There were no significant differences between the Women's CoOp condition and the treatment-as-usual condition in the outcomes presented in Table 2. Although a stronger design would have included a no-treatment control condition to fully assess treatment efficacy, the present study focused primarily on feasibility of the Women's Coop for pregnant African-American women. 

In contrast to the prior examination of the Women's CoOp in non-pregnant African-American women [30], this sample of pregnant women showed no improvement in unemployment. 

However, it is important to note that each of the significant changes from baseline to followup for the entire sample was significant among just women included in the Women's CoOp condition.

### 4.3. Birth Outcomes

Two study participants had miscarriages during the study, one Women's CoOp for Pregnant African-American Women participant and one treatment-as-usual participant, at the gestational ages of 28 and 30 weeks, respectively. The Women's CoOp for Pregnant African-American Women and control conditions both resulted in, on average, term births, 38.2 weeks (SD = 1.9) and 37.2 weeks (SD = 5.8), respectively. The mean birth weight, length, and head circumference also were within normal limits for gestational age in both the Women's CoOp for Pregnant African-American Women and treatment-as-usual conditions.

## 5. Discussion

There are several notable findings from this initial examination of the feasibility of this evidence-based intervention modified for pregnant women. First, this study confirms and emphasizes the ability of outpatient drug-abuse treatment to reduce cocaine and other illegal drug use among pregnant women in an extent similar to non-pregnant patients [32]. These results, coupled with promising birth outcomes, underscore the ability of pregnant women to make meaningful changes in their drug use behavior and serves as evidence that intervention tailored specifically to the needs of pregnant women can be successful. Second, this study suggests that, as with the original Women's CoOp, the Women's CoOp with pregnant African-American women showed reductions in homelessness for its participants. While both the Women's CoOp for Pregnant African-American Women and the treatment-as-usual conditions showed marked improvements in helping women to secure housing only the Women's CoOp for Pregnant African-American Women reduced it to 0%. This reduction in homelessness is similar to that of the Women's CoOp for non-pregnant African-American women [30]. Third, the data from this initial feasibility study of the adaption of the Women's CoOp for pregnant women suggests that this intervention could be successful in teaching women the needed skills to avoid violent situations and/or not use violence against others in situations. Fourth, this study provides valuable information regarding the extent to which these women had unplanned pregnancies that brought them into treatment. The fact that 9 out of 10 women reported an unplanned pregnancy speaks to the dire need for education regarding family planning options, access to these options, and the skills and support to implement their chosen family planning method. Data from this study also demonstrate that pregnant African-American women in drug-abuse treatment continue to have low rates of condom use and are unable to insist on condom use with partners all the time. These results support the need for drug-abuse treatment programs to infuse and integrate HIV sexual-risk reduction messages, education, and skills practice into their programs. Finally, our results, although not definitive, certainly speak to the importance of future research to more fully examine the efficacy of Women's CoOp with pregnant African-American women.

## 6. Study Limitations

The present study is not without its limitations. First, it is an initial feasibility study undertaken to examine the potential utility of the Women's CoOp with pregnant African-American women. Therefore, the impact of the intervention would not be expected to be maximal relative to the success of other Women's CoOp interventions. Second, because 23 of the 96 (24%) women screened for the study were ineligible due to refusal of drug-abuse treatment, the generalizability of the results to the larger pregnant drug-abusing population may be limited; however, this result speaks to the fear, stigma, and concern women have with drug-abuse treatment and the need for future studies to examine ways of changing treatment to be more attractive to these participants. Third, the sample was quite diverse in its use of licit and illicit substances. This diversity may have adversely impacted the efficacy of this adaptation of the Women's CoOp intervention, since the original version of the Women's CoOp targeted crack-abusing African-American women. Fourth, in contrast to other examinations of the Women's CoOp, this study tested the intervention in the context of intensive outpatient drug-abuse treatment. This intense service may have compromised the ability to see improvements specific to the Women's CoOp. Fifth, because of the small sample size, the study may have insufficient power to detect significant changes in some outcomes. Finally, despite that RRBA was revised for the present study, further research establishing its reliability and validity in pregnant African-American women is certainly needed. Nonetheless, results from the current study suggest that future research should examine the Women's CoOp as a potentially efficacious intervention for an underserved population seriously in need of such an intervention. 

## 7. Conclusions

These results demonstrate not only the success of drug-abuse treatment to help improve the risk behaviors and social circumstances of pregnant women but also the potential feasibility of the Women's CoOp to eliminate homelessness. This pregnancy-specific adaption of the Women's CoOp argues for the extension of the reach of this intervention by also suggesting the possibility of reduced violence involvement for its participants. Moreover, these results demonstrate the myriad of stressors these women face in their daily lives and how these issues can compromise treatment seeking and engagement. Finally, these results also support the need for ongoing integration of family planning and HIV sexual-risk reduction into drug-abuse treatment for pregnant African-American women. 

## Figures and Tables

**Table 1:** Baseline demographic and background characteristics for the total sample and the Women's CoOp for Pregnant African-American Women and treatment-as-usual conditions.

	Total sample (*N* = 59)	Women's CoOp (*n* = 30)	Treatment-as-usual (*n* = 29)	*P*
Demographic Characteristics				
	*M* (*SD*)			

Maternal age in years	28.7 (6.6)	28.2 (5.7)	29.2 (7.5)	.76
	*f* (*%*)			
Race: Black/African American	59 (100%)	30 (100%)	29 (100%)	—

Education				
Completed high school	21 (36%)	10 (33%)	11 (38%)	.79

Relationship status				
Never married	48 (81%)	23 (77%)	25 (86%)	.51

Pregnancy				
Unplanned	53 (90%)	27 (90%)	26 (90%)	1.00
Had you gone for prenatal care for this pregnancy?	52 (88%)	28 (93%)	24 (83%)	.25
Used any drugs since you found out pregnant?	41 (70%)	22 (73%)	19 (66%)	.58

Interpersonal violence				
Ever been physically abused:				
Never	44%	40%	48%	.60
More than 12 months ago	31%	33%	28%	.78
One year ago or less	25%	27%	10%	.18
Ever been sexually abused:				
Never	58%	50%	66%	.29
More than 12 months ago	31%	37%	24%	.40
One year ago or less	12%	13%	10%	1.00

The test statistic for maternal age was the independent-samples *t*-test. The remaining variables were tested with the *χ* ^2^ goodness-of-fit test, with *df* = 1. Probability values for the *χ* ^2^ tests are exact. Percentages are within the respective group.

**Table 2:** Outcomes for the six-month reduced sample.

	Differences over time		
	Total sample		
	Baseline	6-month	*P*
Currently homeless	42%	6%	<.001
Currently unemployed	78%	70%	.41

Past 90 days, used			
Cocaine, any form	56%	36%	.03
Use of one or more of illegal drugs (methamphetamine, amphetamine, cocaine, heroin, marijuana, and/or ecstasy)	82%	52%	.001

Reason quit/cut back drugs			
Want to get clean for myself	78%	84%	.18
Wanted to have healthy baby	80%	76%	.16
Child Protective Services would take baby away	56%	50%	.44

What helped you stay clean and sober the most			
Treatment		36%	
Staying away from people (you) used to drink, get high with OR places (you) used to drink, get high OR old neighborhood		38%	

In the past 90 days, one or more times			
Meeting with specialist about relationship with spouse or family member	30%	18%	.16
Meeting with specialist on parenting	46%	38%	.43
Meeting with specialist regaining contact	32%	6%	.002
Meeting with specialist on getting along with children	44%	28%	.1
Meeting focused on regaining contact with children	34%	12%	.01
Any meeting or session	56%	76%	.03

Current alcohol and drug problem			
Have alcohol problem at this time	18%	28%	.23
Have drug problem at this time	58%	62%	.67

Sexual behavior and HIV and prenatal drug use knowledge			
How many days in the past 90 did you have any sex? (Mean (SD))	18 (25)	11 (21)	.13
HIV and prenatal drug risk knowledge: Total score (Mean (SD))	18 (3)	21 (2)	<.001
Condom use in past 90 days	*N* = 21		.51
Never	33%	38%	
Almost never	10%	14%	
Any use	57%	48%	
Can insist on condom use with main sex partner in past 90 days	50% (*N* = 18)	61%	.53

Past 90 days threatened any violence			
Threatened any violence	62%	34%	.003

*N* = 50 due to missing data at 6-month followup. The tests of the differences over time are based on the McNemar test for matched data, with the exception of the tests for condom use, which are based on the Wilcoxon signed-rank test, and the number of days in the past 90 days on which the respondent had any sex and the HIV knowledge: total score, which are based on the paired *t*-test.
